# The Octopamine Receptor Octβ2R Regulates Ovulation in *Drosophila melanogaster*

**DOI:** 10.1371/journal.pone.0104441

**Published:** 2014-08-06

**Authors:** Junghwa Lim, Paul R. Sabandal, Ana Fernandez, John Martin Sabandal, Hyun-Gwan Lee, Peter Evans, Kyung-An Han

**Affiliations:** 1 Department of Biological Sciences, Border Biomedical Research Center/Neuroscience and Metabolic Disorders, University of Texas at El Paso, El Paso, Texas, United States of America; 2 The Inositide Laboratory, The Babraham Institute, Cambridge, United Kingdom; Lancaster University, United Kingdom

## Abstract

Oviposition is induced upon mating in most insects. Ovulation is a primary step in oviposition, representing an important target to control insect pests and vectors, but limited information is available on the underlying mechanism. Here we report that the beta adrenergic-like octopamine receptor Octβ2R serves as a key signaling molecule for ovulation and recruits protein kinase A and Ca^2+^/calmodulin-sensitive kinase II as downstream effectors for this activity. We found that the *octβ2r* homozygous mutant females are sterile. They displayed normal courtship, copulation, sperm storage and post-mating rejection behavior but were unable to lay eggs. We have previously shown that octopamine neurons in the abdominal ganglion innervate the oviduct epithelium. Consistently, restored expression of Octβ2R in oviduct epithelial cells was sufficient to reinstate ovulation and full fecundity in the *octβ2r* mutant females, demonstrating that the oviduct epithelium is a major site of Octβ2R’s function in oviposition. We also found that overexpression of the protein kinase A catalytic subunit or Ca^2+^/calmodulin-sensitive protein kinase II led to partial rescue of *octβ2r*’s sterility. This suggests that Octβ2R activates cAMP as well as additional effectors including Ca^2+^/calmodulin-sensitive protein kinase II for oviposition. All three known beta adrenergic-like octopamine receptors stimulate cAMP production *in vitro*. Octβ1R, when ectopically expressed in the *octβ2r*’s oviduct epithelium, fully reinstated ovulation and fecundity. Ectopically expressed Octβ3R, on the other hand, partly restored ovulation and fecundity while OAMB-K3 and OAMB-AS that increase Ca^2+^ levels yielded partial rescue of ovulation but not fecundity deficit. These observations suggest that Octβ2R have distinct signaling capacities *in vivo* and activate multiple signaling pathways to induce egg laying. The findings reported here narrow the knowledge gap and offer insight into novel strategies for insect control.

## Introduction

Mating triggers comprehensive physiological and behavioral changes in female insects to maximize reproductive success. Notably, sex peptide, a seminal fluid protein transferred during copulation, activates oviposition, enhances locomotor activity, decreases sexual receptivity, shortens daytime sleep, and alters immunity and food choice in *Drosophila melanogaster* [1]–[4]. While broadly present in the reproductive, endocrine and nervous systems, the sex peptide receptor expressed in the fruitless, pickpocket and doublesex neurons in particular plays a central role in reducing sexual receptivity and increasing oviposition processes that directly and substantially contribute to fecundity [5]–[8]. Information regarding the downstream effectors and signaling pathways, however, is largely unknown. Enhanced understanding of the molecules and target sites mediating individual post-mating processes is needed to narrow the knowledge gap and gain insights into an effective strategy to control female fecundity.

Oviposition (egg-laying) consists of ovulation, transfer of a mature egg from the ovary to the uterus where fertilization occurs, and deposition of eggs to an external location with proper environmental conditions. Octopamine (OA), a major biogenic amine in insects, is vital for oviposition [9]–[14]. Females lacking the vesicular monoamine transporter (VMAT), which is involved in storage and exocytotic release of biogenic amines, are sterile and the sterility is rescued by transgenic VMAT expression in the OA but not in other biogenic amine neurons in the *vmat* mutant [15]. OA is made from tyrosine by the sequential actions of tyrosine decarboxylase (TDC) and tyramine beta-hydroxylase (TβH), and functions as a neurotransmitter, neuromodulator and neurohormone [16]. Similar to the *vmat* mutant, the *tdc2* or *tβh* mutant females are sterile, and OA feeding in the mated *tdc2* or *tβh* females is sufficient to induce egg-laying [11], [14]. While OA neurons have broad projection patterns within and outside of the central nervous system (CNS) [17], [18], the subset of OA neurons in the abdominal ganglion that innervates the reproductive system plays a key role in oviposition since restored TβH expression in those neurons reinstates fecundity of the *tβh* females [10]. In the reproductive system OA axon terminals are found in the ovaries, oviducts, sperm storage organs and uterus where OA is likely to exert multiple functions [10]–[13]. For instance OA, when applied to the dissected reproductive system, modulates muscle activity in a tissue specific manner: it enhances muscle contractions in the ovary but inhibits them in the oviduct [12], [13]. This suggests that OA receptors present in the ovary, oviduct and other areas regulate distinct elements of the reproductive process.

Five G-protein coupled receptors specific for OA are identified in *Drosophila* and comprise two alpha1 adrenergic-like receptors OAMB-K3 and OAMB-AS generated from the *oamb* locus by alternative splicing and three beta adrenergic-like receptors Octβ1R, Octβ2R and Octβ3R [19]–[21]. When assayed in cultured cells heterologously expressing these receptors, the alpha-1-like OAMB stimulates the increase in intracellular Ca^2+^ whereas the beta-like receptors increase cAMP levels [19]–[21]. We have previously shown that OAMB in the oviduct epithelium is involved in mediating the octopaminergic signal for ovulation [22]. In this report, we demonstrate that the oviduct epithelium also requires an additional OA receptor, the beta-like Octβ2R, for ovulation and full fecundity. We also show that the downstream effectors of Octβ2R and OAMB have non-overlapping functions in the oviduct epithelium for fecundity.

## Materials and Methods

### *Drosophila* strains and culture

All flies, unless otherwise stated, were raised in the standard yeast/cornmeal/agar medium at 25°C with 50% relative humidity and on a 12-h light-dark cycle. *Canton-S* (*CS*) was used as a wild-type strain. The *octβ2r* mutant used in this study is the transgenic line *octβ2r^f05679^* generated by the Gene Disruption Project [23], [24]. *octβ2r^f05679^* contains the piggyBac transposon inserted in the 5^th^ exon of the *octβ2r* gene in the third chromosome, interrupting the coding sequence [24], thus likely represents a hypomorphic or possibly null allele [25]. *octβ2r^f05679^* (hereafter *octβ2r*) was obtained from the Bloomington Stock Center (stock no. 18896) and backcrossed with Cantonized *w^1118^* for six generations. The *oamb* mutant used in this study is the null allele *oamb^286^* [9]. *Heat shock* (*HS)-GAL4* (stock no. 2077), *elav-GAL4* (stock no. 8765), *UAS-CaMKII-R3* (stock no. 29662) and *don juan (dj)-GFP* (stock no. 5417) flies were obtained from the Bloomington Stock Center, *UAS-PKAc* from Dr. Kalderon (Columbia University, New York, NY) and *nSyb-GAL4* from Dr. Ordway (Pennsylvania State University, State College, PA). The *RS-GAL4, UAS-OAMB-K3 and UAS-OAMB-AS* lines are described in Lee et al. [22], [26]. Individual transgenes (*HS-GAL4, elav-GAL4, nSyb-GAL4, RS-GAL4, UAS-OAMB, UAS-PKAc and UAS-CaMKII-R3*) were placed in the *octβ2r* mutant background for rescue experiments.

### UAS-OctβR transgenic flies

Octβ1R, Octβ2R, and Octβ3R cDNAs containing the open reading frame [20] were cloned under UAS in the gateway vector pTW [27]. In addition, Octβ2R was cloned in pTWG, which allows GFP to be fused to the C-terminus of Octβ2R, for monitoring receptor expression and localization. The cloned receptors were injected into *w^1118^* embryos, and germ-line transformed lines were outcrossed with Cantonized *w^1118^* for six generations to normalize the genetic background and cross out potential second site mutations. The transgenes were then placed in the *octβ2r* mutant genetic background for rescue experiments.

### Fecundity tests

For ovulation analysis, virgin females were collected within 12 h after eclosion and aged for 4 to 5 days before tests. Ten virgin females were placed with thirty *CS* males in a food vial for 18 h for mating and then were anaesthetized on ice. The female reproductive system was dissected to determine the presence of an egg in the lateral or common oviduct, or uterus. The percentage of females with an egg per vial was used as one data point. In the experiments involving HS-GAL4, the control and transgenic *octβ2r* mutant females reared at room temperature were treated with heat shock at 37°C for 30 min twice with a 5 h interval. After 4 h of recovery at room temperature, they were subjected to mating and ovulation tests. For progeny counts, three virgin females and six *CS* males were placed in a food vial for three days and then removed. The number of progeny was counted 14 days later. In sperm retention analysis, *CS* or *octβ2r* virgin females were mated with *dj-GFP* males, in which sperm is tagged with GFP. The female reproductive system was dissected 24 or 48 h later and processed as described in the Histological analysis section below.

### Behavioral tests

For courtship, copulation and receptivity analyses, 4 day-old *CS* or *octβ2r* virgin females were individually paired with *CS* males in a courtship chamber and videotaped to score courtship activity, copulation initiation time and copulation duration [9], [28]. The percentage of time that a male spent courting a female during the first 10 min of pairing was used as courtship index (CI). In receptivity tests, the females mated with *CS* males were gently transferred to a food vial and housed alone. After 48 h they were individually paired with naïve *CS* males and videotaped to measure courtship and copulation activities.

### Histological analysis

The female reproductive system that includes the ovary, oviduct, uterus, sperm storage organs, and accessory glands was dissected in phosphate buffered saline (PBS) and fixed in PBS containing 4% paraformaldehyde for 20 min at room temperature [22]. For cryosections, whole female flies were fixed in PBS containing 4% paraformaldehyde and 40 mM lysine for 3 h and soaked in 25% sucrose solution overnight at 4°C. Ten micron sagittal sections were made and placed on a Superfrost microscope slide (Thermo Fisher Scientific, Waltham, MA). The dissected and cryosectioned tissues were then washed with PBS and 0.12 M Tris-HCl, pH 7.4, three times for 10 min each and mounted in the Vectashield mounting medium containing DAPI (Vector Labs, Burlingame, CA). Images were collected using the Zeiss LSM 700 confocal microscope (Carl Zeiss, Thornwood, NY) and processed using the Image J software (NIH).

### RNA analysis

The female reproductive system was dissected as mentioned above and ovaries were taken out to enrich RNA from the oviduct. Fifty dissected tissues were pooled and homogenized in 10 µl of the lysis buffer RLT (Qiagen, Valencia, CA) with the Kontes micro tissue grinder (Thermo Fisher Scientific) followed by the QIAshredder spin column (Qiagen). Total RNA was extracted using RNeasy Protect Mini kit (Qiagen) and cDNA was synthesized using SuperScript III First-Strand Synthesis System (Invitrogen, Carlsbad, CA) for PCR. Quantitative PCR (qPCR) was performed and analyzed using the iQ SYBR Green Supermix kit (Bio-Rad, Hercules, CA) in the MyIQ Single-Color Real-Time PCR detection system (Bio-Rad) according to the manufacturer’s instructions. Twenty to 100 ng of cDNA samples were run in triplicates and the reactions were done with two different primer sets for individual receptors and ribosomal protein L32 (Rp49; [29], [30]), which was used as a reference gene to normalize receptor expression levels. All primer sets were designed to span at least one intron and checked for specificity using the Flybase Blast against the *Drosophila* genome [24]. The PCR primers were: for Octβ1R, F1-TGTGCAGCCACTGGACTATC, R1-TATGGCGTATGCCTTGTTCA, F2-AGCATCATGCACCTCTGTTG, R2-GTGTACCATCCCGAGCAGAT; for Octβ3R, F1-ATTTCAGTGCAGCGCAATC, R1-CATCCAGGCTGTTGTACACG, F2-TTCCACGTTTGAGCTCCTCT, R2-GCCAGCGACACAACAAAGTA; for Rp49, F1-TACAGGCCCAAGATCGTGAA, R1-GTTCGATCCGTAACCGATGT, F2-CGCACCAAGCACTTCATCC, R2-AGCGGCGACGCACTCTGT.

### Data analysis

Statistical analyses were performed using Minitab 16 (Minitab, State College, PA) and JMP 10 (SAS, Cary, NC). All data are presented as mean ± SEM and normality was determined by the Anderson Darling goodness-of-fit test. Normally distributed data were analyzed with Student’s *t*-test or ANOVA and *post hoc* Tukey–Kramer tests while non-normally distributed data typically observed in courtship indices or fecundity data with many values close to zero were analyzed by Kruskal-Wallis and *post hoc* Mann-Whitney tests.

## Results

### The sterility phenotype of the *Octβ2r* mutant female

**Figure 1:**
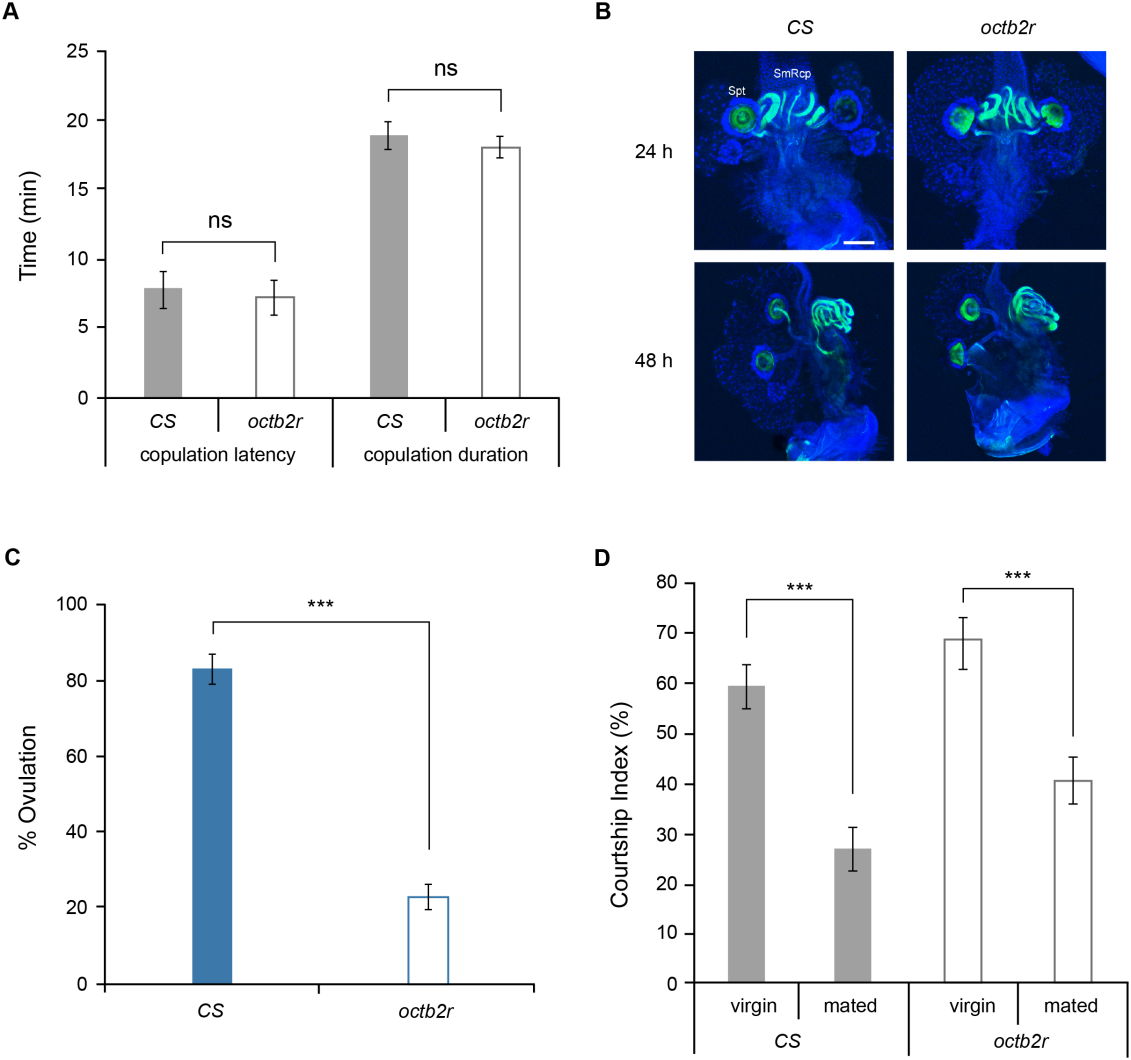
The sterility phenotype of the *octβ2r* mutant female is due to defective ovulation. (A) Copulation behavior. Virgin wild-type *CS* or *octβ2r* females were singly paired with naïve *CS* males to examine copulation behavior. The *CS* males paired with *octβ2r* females exhibited copulation latency and duration comparable to those paired with *CS* females (*p*>0.05 by Student’s t-test; *n* = 17–24). (B) Sperm retention. *CS* and *octβ2r* females were mated with *dj-GFP* males carrying GFP-tagged sperm (green) and the reproductive systems were dissected and counterstained with DAPI (blue). *CS* and *octβ2r* females had comparable sperm storage in the seminal receptacle and spermathecae at 24 and 48 h after mating. Spt, spermathecae; SmRcp, seminal receptacle. Scale bar, 50 µm. (C) Ovulation. *CS* and *octβ2r* females were mated with *CS* males and the reproductive systems were dissected to examine the location of the egg in the oviduct or uterus. The *octβ2r* females had significantly lower levels of ovulation than *CS* females (***, *p*<0.0001 by Student’s t-tests; *n* = 10). (D) Courtship behavior. Virgin *CS* and *octβ2r* females were singly paired with naïve *CS* males to measure courtship activity. The mated females were tested again with new naïve males 48 h later to measure courtship receptivity. The percentage of time that the CS males spent courting *CS* or *octβ2r* females represents courtship index. With both *CS* and *octβ2r* females, courtship activities of the males paired with mated females were significant lower than those of the males paired with virgin females (*p*<0.0001 by Mann-Whitney; *n* = 32–69). Thus, *CS* and *octβ2r* females have comparable pre- and post-mating courtship activity. ns, not significant.

When the *octβ2r* homozygous mutant flies were housed together, no progeny were detectable. To determine whether females or males contribute to sterility, *octβ2r* mutant males or females were placed with *CS* females or males, respectively. While the *octβ2r* males were fertile (data not shown), the *octβ2r* females did not produce any progeny. Female fecundity is affected by several behavioral and physiological factors. For example, failure to court or copulate with a male, retain sperm or ovulate and deposit eggs would lead to sterility. When tested with *CS* males, the *octβ2r* females had copulation latency and duration times comparable to those of *CS* females (*p*>0.05; Figure 1A). To examine sperm retention, the *octβ2r* females were mated with *dj-GFP* males, in which sperm is labeled with GFP. At 24 and 48 h after mating, no anomalies were detectable in the sperm stored in the sperm storage organs, seminal receptacle and spermathecae (Figure 1B). Also, there was no ectopically located sperm in other areas of the *octβ2r* reproductive system. When the reproductive system was examined for ovulation activity, on the other hand, a substantially lower percentage of *octβ2r* females had an egg in the oviduct or uterus at 18 h post mating compared to *CS* females (*p*<0.0001; Figure 1C). This suggests that impaired ovulation is responsible for the *octβ2r*’s sterility phenotype.

Ovulating female flies are reluctant to re-mate and thus show rejection behavior to courting males, leading to decreased courtship activity by the rejected males. Previous studies show that the females defective in ovulation are also impaired in post-mating rejection behavior [1], [5]–[8]. We investigated whether the *octβ2r* mutant females have similar phenotypes by testing their courtship activity with *CS* males. As shown in Figure 1D, *CS* males exhibited significantly less courtship activity with mated *CS* females than with virgin *CS* females as predicted (*p*<0.0001). Likewise, *CS* males paired with mated *octβ2r* females showed reduced courtship compared to those paired with virgin *octβ2r* females (*p*<0.0001) and none of the mated *octβ2r* females were engaged in copulation. When *CS* males’ courtship activities toward virgin *CS* vs. *octβ2r* females or mated *CS* vs. *octβ2r* were examined, no difference was observed (Figure 1D, *p*>0.05), supporting that the *octβ2r* females have normal courtship and rejection behavior. Taken together, these observations indicate that Octβ2R is essential for ovulation but dispensable for pre- and post-mating behaviors.

### Octβ2R’s functional site in ovulation

**Figure 2:**
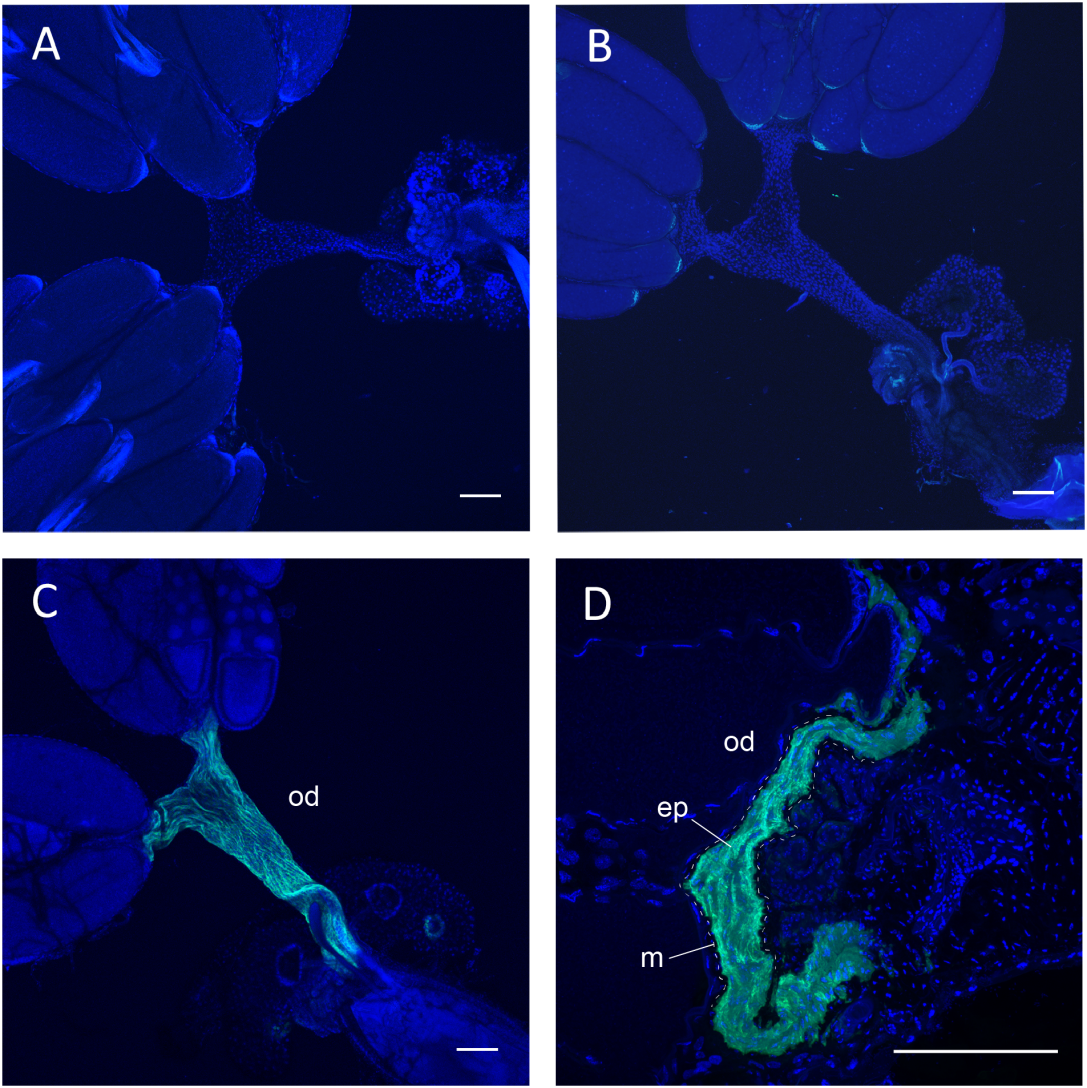
Octβ2R expression in the oviduct epithelium. (A) *CS.* (B) *octβ2r* carrying *UAS-Octβ2R-GFP*. (C, D) *octβ2r* carrying *RS-GAL4* and *UAS-Octβ2R-GFP.* The whole mount (A–C) and cryosectioned (D) female reproductive systems were counterstained with DAPI (blue). Octβ2R-GFP (green) expression is clearly visible in the oviduct epithelium, but not in other areas, of the *octβ2r* female carrying *RS-GAL4* and *UAS-Octβ2R-GFP* (C, D). od, oviduct; ep, oviduct epithelial layer; m, oviduct muscle layer demarcated with dashed lines. Scale bars, 100 µm.

**Figure 3:**
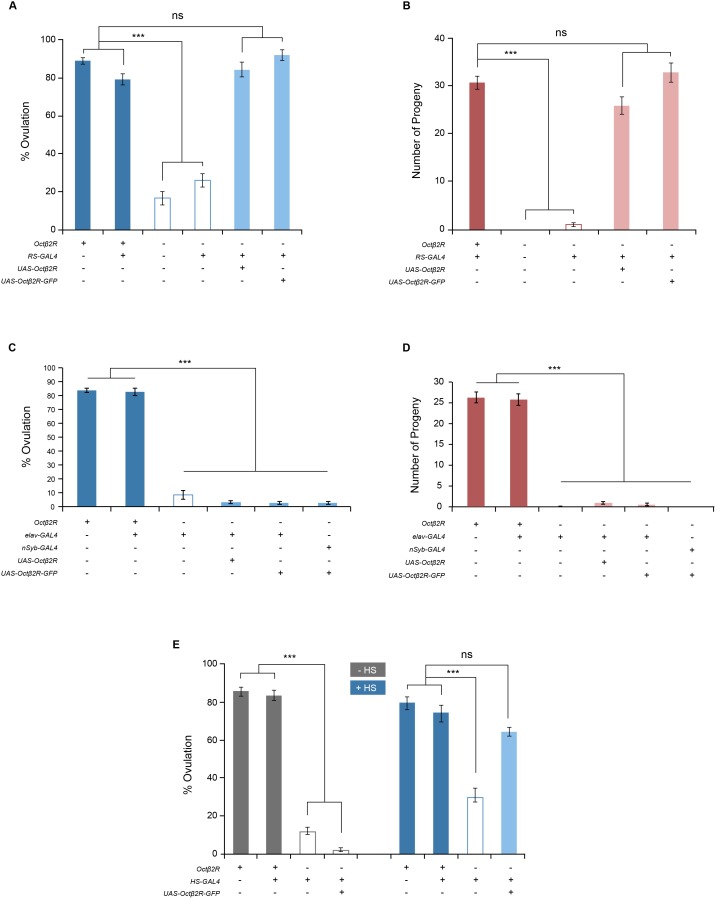
Restored Octβ2R expression in the oviduct epithelium rescues the *octβ2r’s* ovulation and fecundity phenotypes. (A, B) Epithelial rescue. The *octβ2r* females carrying *RS-GAL4* and either *UAS-Octβ2R* or *UAS-Octβ2R-GFP* had ovulation (A) and fecundity (B) levels comparable to those of *CS* females (ns, *p*>0.05; ***, *p*<0.0001; *n* = 17–24 for ovulation tests; *n* = 18–39 for fecundity tests. (C, D) Neuronal rescue. The *octβ2r* females carrying the pan-neural driver elav-GAL4 or nSyb-GAL4 along with either *UAS-Octβ2R* or *UAS-Octβ2R-GFP* exhibited ovulation (C) and fecundity (D) levels comparable to those of the *octβ2r* mutant females. ***, *p*<0.0001; *n* = 12–27 for ovulation tests; *n* = 15–37 for fecundity tests. (E) Temporal rescue. The females reared at the non-permissive temperature were subjected to heat shock (+HS) to induce Octβ2R expression or kept at non-permissive temperature (−HS) as a no-induction control. The *octβ2r* females carrying *HS-GAL4* and *UAS-Octβ2R-GFP* treated with heat shock displayed ovulation levels similar to *CS* (ns, *p*>0.05; *n* = 13–30) while the females of the same genotype without heat shock showed the ovulation levels significantly different from *CS* females (***, *p*<0.0001). In the graph table, “+” denotes the presence of one normal *octβ2r* allele (heterozygous *octβ2r*) or a single copy of transgenes except for the first row, which represents wild-type *CS* with two normal *octβ2r* alleles, and “−“ denotes the absence of normal *octβ2r* alleles or transgenes.

OA containing axons are present in the CNS as well as the reproductive system, thus the site of the OA receptor Octβ2R’s function in ovulation could be the CNS neurons controlling ovulation or the reproductive tissue directly involved in ovulation. The microarray and RNA-seq analyses [24] show that Octβ2R is expressed at very low levels in the CNS and reproductive tissue, which we also observed with quantitative RT-PCR (data not shown). We have previously shown that OA neurons in the abdominal ganglion innervate the oviduct epithelium where alpha1 adrenergic-like OAMB is involved in ovulation [22]. To identify the site of the Octβ2R’s action, we adopted the GAL4/UAS binary system, in which the transcription factor GAL4 binds to UAS to activate the downstream gene expression [31]. For tissue-specific expression we used pan-neuronal drivers elav-GAL4 and nSyb-GAL4, and the reproductive system driver RS-GAL4 that has no neuronal expression [22]. We also employed the fusion construct Octβ2R-GFP, in which GFP is fused to the C-terminus of Octβ2R, to monitor the site and level of transgene expression. When driven by RS-GAL4, Octβ2R-GFP was conspicuously visible in the oviduct epithelium but not in the oviduct muscle and ovaries (Figure 2). The transgenic *octβ2r* females with *RS-GAL4* and *UAS-Octβ2R* or *UAS-Octβ2R-GFP* exhibited ovulation and fecundity to the levels significantly different from the *octβ2r* females (*p*<0.0001) but comparable to those of the control females (*p*>0.05; Figure 3A and 3B). On the contrary, the *octβ2r* females carrying the neuronal driver *elav-GAL4* and *UAS-Octβ2R* or *UAS-Octβ2R-GFP* showed the similar ovulation and fecundity levels as the *octβ2r* females (*p*>0.05) and the same results were obtained when another neuronal driver or *nSyb-GAL4* was used (Figure 3C and 3D). These observations indicate that the oviduct epithelium is a critical site of the Octβ2R’s function in ovulation and the nervous system is spared in this process.

We next explored whether Octβ2R is involved in a developmental or physiological process for ovulation. We used HS-GAL4, in which GAL4 expression is controlled by the heat-inducible *hsp70* promoter [24], to induce Octβ2R expression upon simple temperature shift. The *octβ2r* females carrying *HS-GAL4* and *UAS-Octβ2R-GFP* as well as the control females were subjected to heat treatment at 37°C (Figure 3E, +HS) and then allowed to mate with *CS* males at room temperature. Another group of females with the same genotypes that did not receive heat treatment (−HS) was used as an uninduced control. When tested for ovulation, the *octβ2r* females with *HS-GAL4* and *UAS-Octβ2R-GFP* showed a heat-shock dependent rescue of the ovulation phenotype (Figure 3E). This study suggests a physiological, rather than developmental, role of Octβ2R in ovulation.

### Downstream effectors of Octβ2R

**Figure 4:**
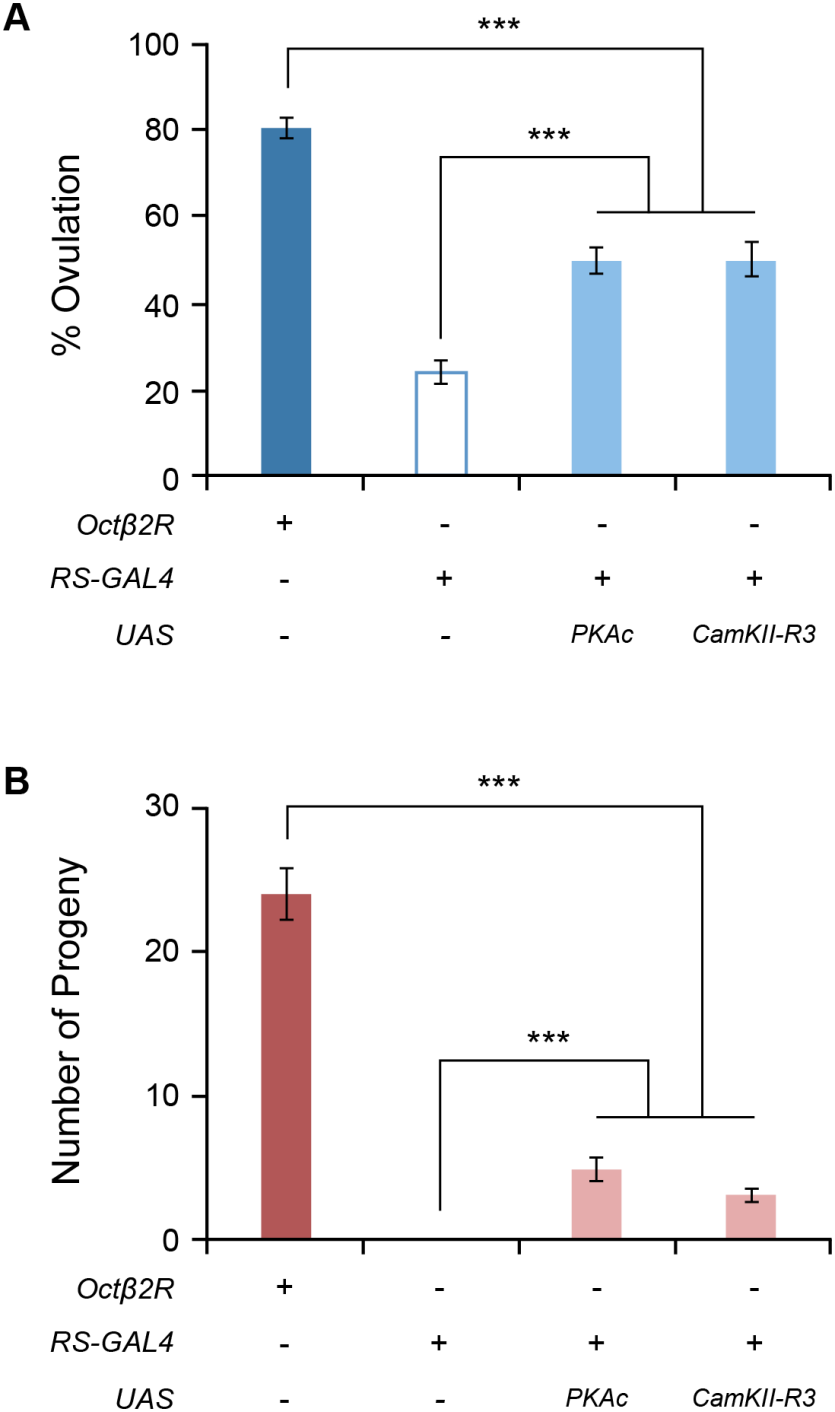
Octβ2R activates both PKA and CaMKII in the oviduct epithelium for ovulation and fecundity. Ectopic expression of PKAc or CaMKII-R3 was induced with RS-GAL4 in the oviduct epithelium of the *octβ2r* mutant females. The *octβ2r* females carrying RS-GAL4 along with UAS-PKAc or UAS-CaMKII-R3 partially rescued the *octβ2r*’s ovulation (A) and fecundity (B) phenotypes (***, *p*<0.0001, *n* = 21–26 for ovulation tests, *n* = 21–38 for fecundity tests).

Octβ2R is a G-protein coupled receptor. Thus, a physiological role for Octβ2R after binding to OA is likely to involve the activation of intracellular signaling pathways, which in turn trigger epithelial cell activity facilitating egg delivery from the ovary to the uterus. In an effort to elucidate the cellular mechanism responsible for this activity, we have investigated downstream molecules mediating Octβ2R’s effect on ovulation. In this study, we focused on the protein kinases that functionally interact with Octβ2R in the oviduct epithelium. As noted, Octβ2R stimulates cAMP production in transfected cells, making cAMP-dependent protein kinase A (PKA) an excellent candidate to serve as a downstream effector of Octβ2R. PKA activity is normally repressed in the absence of cAMP since the catalytic subunit is bound to the regulatory subunit. Upon binding of cAMP to the regulatory subunit, the catalytic subunit is released to act on its substrates. If PKA is indeed a downstream effector of Octβ2R in the oviduct epithelium, we reasoned that activation of PKA in a cAMP-independent manner would bypass Octβ2R and stimulate ovulation in the *octβ2r* mutant females. To induce cAMP-independent PKA activation, we overexpressed the catalytic subunit of PKA (PKAc) [32]. Consistent with the notion, the *octβ2r* females overexpressing PKAc in the oviduct epithelium had significantly higher levels of ovulation (*p*<0.0001) and fecundity (*p*<0.0001) than those of the *octβ2r* females (Figure 4A and 4B). The levels, however, were significantly lower than those in the control *CS* females (*p*<0.0001), indicating incomplete rescue. These data corroborate PKA as a key downstream effector of Octβ2R in the oviduct epithelium. Incomplete rescue could be due to an insufficient transgene level or an additional effector(s) required for successful ovulation.

**Figure 5:**
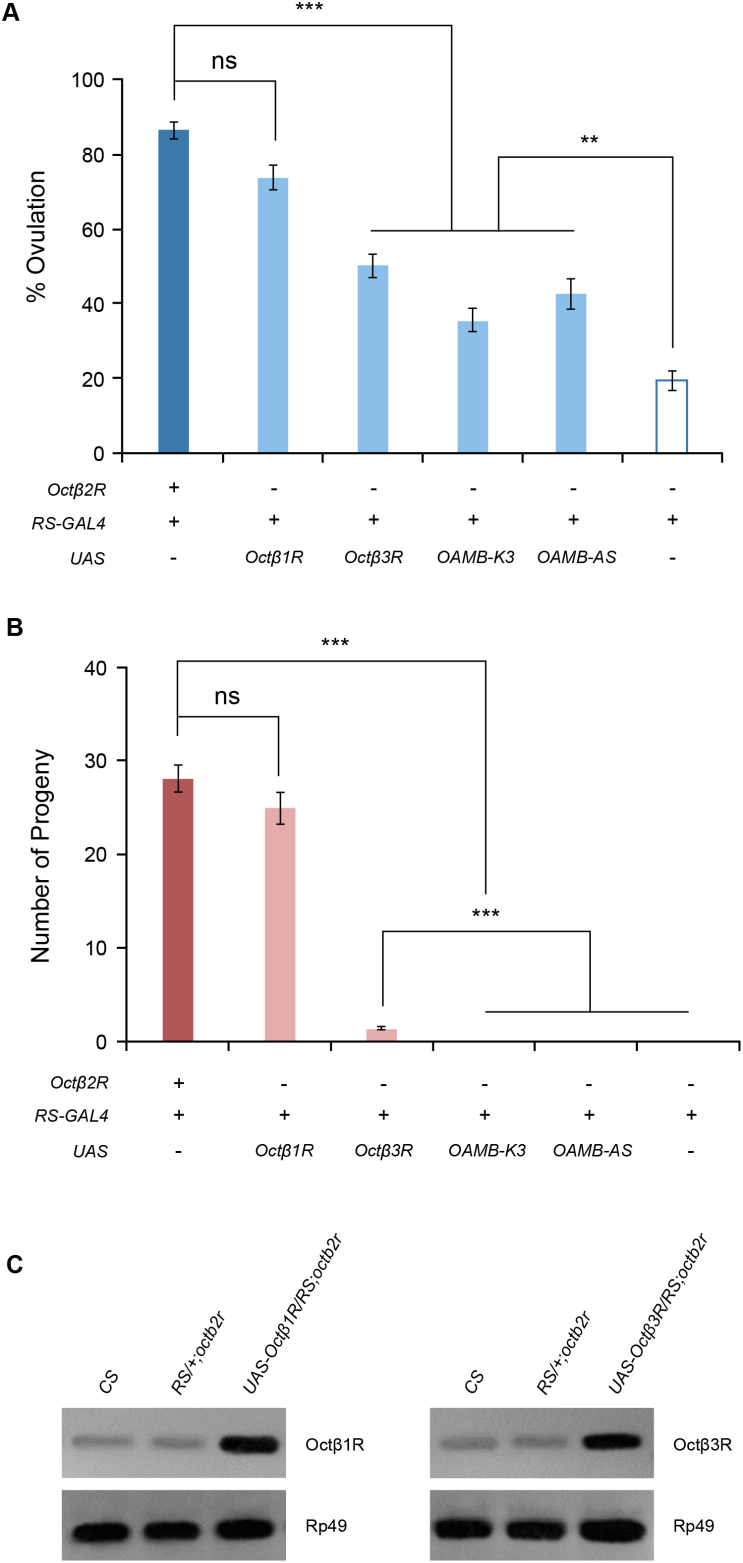
Functional substitution by other OA receptors. Transgenic expression of the OA receptors Octβ1R, Octβ3R, OAMB-K3 and OAMB-AS was driven by RS-GAL4 in the *octβ2r*’s oviduct epithelium. (A) Ovulation rescue. Ectopically expressed *Octβ1R* fully rescued the *octβ2r*’s ovulation phenotype while *Octβ3R*, *OAMB-K3* and *OAMB-AS* yielded partial rescue (***, *p*<0.0001; **, *p*<0.005; ns, not significant; *n* = 18–34). (B) Fecundity rescue. Ectopic expression of *Octβ1R* fully restored fecundity whereas partial or no rescue was observed with ectopic expression of Octβ3R or OAMB-K3 and OAMB-AS, respectively (***, *p*<0.0001, *n* = 20–38). (C) RT-PCR analysis. RNA was isolated from dissected reproductive tissues of *CS*, *octβ2r* mutant females carrying RS-GAL4 alone, and *octβ2r* mutant females carrying RS-GAL4 and UAS- Octβ1R or UAS-Octβ3R for RT-PCR. The elevated levels of Octβ1R or Octβ3R PCR products were detectable in the *octβ2r* females carrying RS-GAL4 and UAS- Octβ1R or UAS-Octβ3R, respectively. Rp49 was used for an internal control.

Ca^2+^/calmodulin-sensitive protein kinase II (CaMKII) is important for ovulation since inhibition of CaMKII decreases ovulation [22]. We tested whether CaMKII acts downstream of Octβ2R to regulate ovulation. Ectopic expression of the CaMKII-R3 isoform in oviduct epithelial cells resulted in partially restored ovulation and fecundity in the *octβ2r* females to the levels comparable to those of PKAc overexpression (Figure 4A and 4B). This suggests that CaMKII may serve as an additional downstream effector of Octβ2R. Conversely, overexpressed CaMKII may induce an alternative pathway to compensate for deficient Octβ2R signaling. To test this, we investigated whether alpha1-like OAMB receptors, which activate CaMKII in the oviduct epithelium, could rescue the *octβ2r*’s sterility phenotype. When ectopically expressed in the oviduct epithelium of the *octβ2r* female, both OAMB-K3 and OAMB-AS reinstated ovulation to a limited extent like overexpressed PKAc or CaMKII-R3 (Figure 5A) but did not rescue fecundity (Figure 5B). These observations suggest that reinstated ovulation conferred by overexpressed CaMKII may be attributable to compensatory activity of the OAMB pathway; on the contrary, rescued fecundity conferred by overexpressed CaMKII is likely independent of the OAMB signaling.

### Genetic interaction of Octβ2R and OAMB

**Figure 6:**
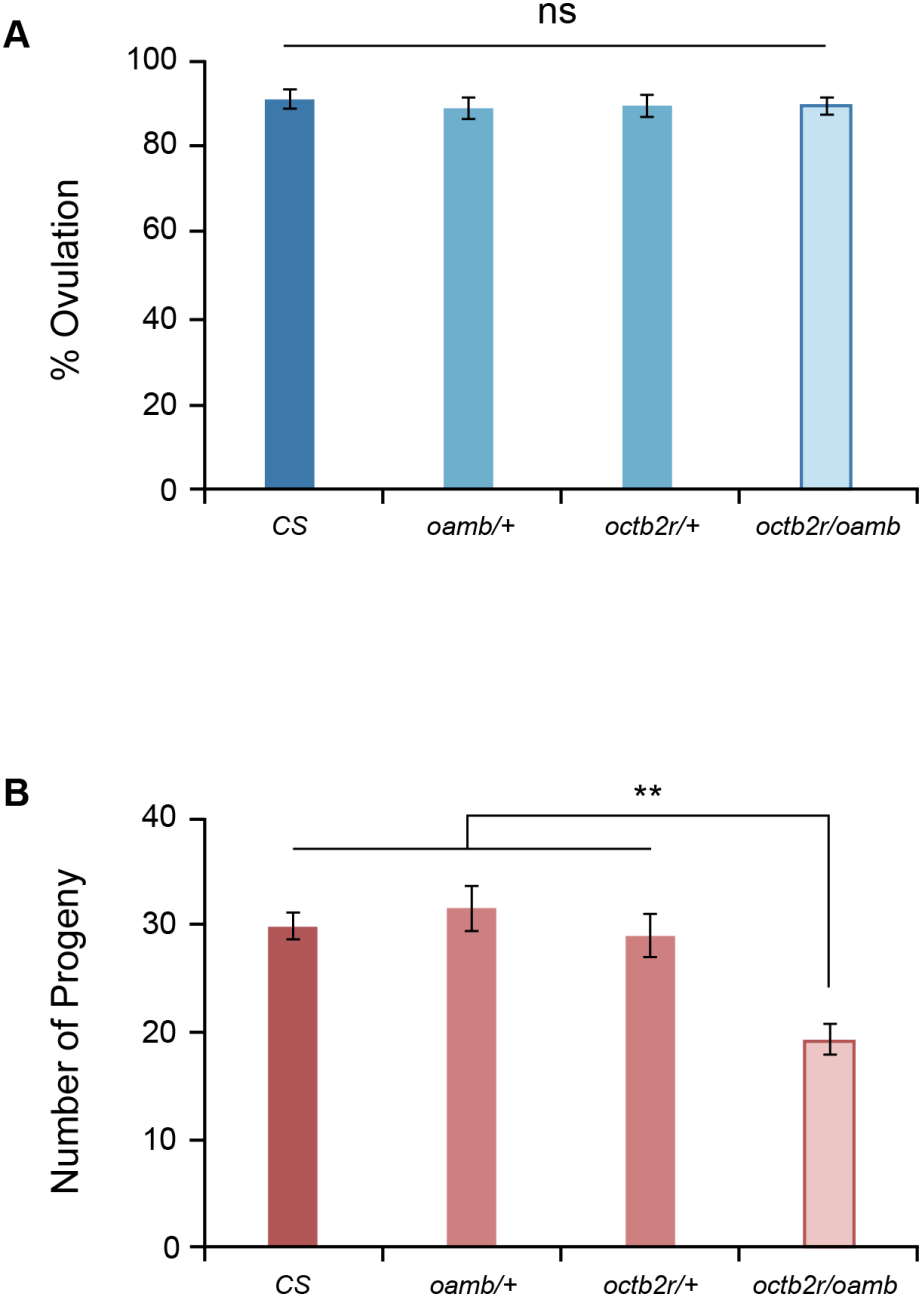
Genetic interaction. The heterozygous *octβ2r* and *oamb^286^* mutant females having two normal alleles of *oamb* and octβ2r, respectively, and the transheterozygous *octβ2r/oamb^286^* females were tested for ovulation (A) and fecundity (B). While the heterozygous *octβ2r* and *oamb^286^* females showed normal ovulation and fecundity, the transheterozygous *octβ2r/oamb^286^* females exhibited ovulation comparable to *CS* (A; ns, *p*>0.05; *n* = 20) but significantly reduced fecundity (*p*<0.001; *n* = 21–22).

At present, there is no information available regarding which downstream molecules of PKA and CaMKII or other effectors of Octβ2R and OAMB mediate ovulation and fecundity. As noted a homozygous mutation in either *octβ2r* or *oamb* causes sterility (this study and [9]), thus both Octβ2R and OAMB signals are required for molecular and cellular activities essential for female fertility. In order to elucidate relative contributions of Octβ2R and OAMB signaling to ovulation and fecundity, we examined the genetic interaction of *octβ2r* and *oamb* heterozygous mutations. The heterozygous *octβ2r* or *oamb* mutant females with normal *oamb* or *octβ2r* alleles, respectively, exhibited the ovulation and fecundity levels comparable to those of *CS* females (*p*>0.05, Figure 6A and 6B). The females with heterozygous mutations in both *octβ2r* and *oamb* also had normal ovulation (Figure 6A) but reduced fecundity (*p*<0.001, Figure 6B). These data suggest that the downstream molecules of Octβ2R and OAMB critical for ovulation are somewhat redundant or overlapping, which is consistent with the result that overexpressed OAMB partly compensates for deficient Octβ2R signaling for ovulation (Figure 5A). On the other hand, the downstream molecules of Octβ2R and OAMB likely have non-redundant or dosage-sensitive functions for fecundity. This is in line with the fact that homozygous mutations in either *octβ2r* or *oamb* cause sterility.

### Functional substitution by other OA receptors

*Drosophila melanogaster* has two additional beta adrenergic-like receptors Octβ1R and Octβ3R and they activate increases in cAMP levels when assayed in heterologous cell lines [20]. There is no information regarding their signaling properties *in vivo*. We hypothesized that Octβ1R and Octβ3R would functionally substitute Octβ2R if they have similar signaling capacities *in vivo*. To test this, we generated the *octβ2r* females carrying *RS-GAL4* along with *UAS-Octβ1R* or *UAS-Octβ3R* and examined them for fertility. Ectopically expressed Octβ1R in the oviduct epithelium fully reinstated ovulation and fecundity in the *octβ2r* females (*p*<0.0001, Figure 5A and 5B). However, Octβ3R yielded only partial restoration of ovulation and fecundity to the levels significantly higher than those of *octβ2r* (*p*<0.0001) but lower than those of control females (*p*<0.0001, Figure 5A and 5B). In transfected cells, Octβ1R and Octβ3R have a higher or similar potency, respectively, compared to Octβ2R in stimulating cAMP increases [20] while their capacities to activate CaMKII are unknown. Different efficacies of Octβ1R, Octβ3R and OAMB-K3 to rescue the *octβ2r* phenotypes could be due to distinct signaling capacities of the OA receptors *in vivo*. Alternatively, expression levels of the transgenic receptors driven by RS-GAL4 could be insufficient to provide full rescue. RS-GAL4 is a strong driver since RS-GAL4-induced GFP, OAMB-AS, OAMB-K3 or Octβ2R-GFP expression is readily detectable and present at high levels in the oviduct epithelium ([22] and Figure 2). Nonetheless, it is possible that RS-GAL4-driven Octβ3R expression may not be sufficient to fulfill Octβ2R’s function deficient in the *octβ2r* females. Since Octβ1R and Octβ3R antibodies are unavailable, we performed RT-PCR to examine transcript levels in the reproductive system of the *octβ2r* females carrying *RS-GAL4* along with *UAS-Octβ1R* or *UAS-Octβ3R*. As shown in Figure 5C, Octβ1R and Octβ3R RNAs were found in the *CS* and *octβ2r* reproductive tissues and present at elevated levels in *RS-GAL4/UAS-Octβ1R;octβ2r* and *RS-GAL4/UAS-Octβ3R;octβ2r, respectively*. We then examined relative abundance by real time RT-PCR using two different primer sets for each receptor. Octβ1R and Octβ3R transcript levels in reproductive tissues of the *octβ2r* females carrying RS-GAL4 and UAS-Octβ1R or UAS-Octβ3R were 21.9±2.3 and 3.2±0.7 folds higher than those in the *octβ2r* reproductive tissue, respectively. The lower level of Octβ3R compared to Octβ1R transcripts may explain partial rescue. It remains to be resolved whether protein levels of the receptors correspond to the mRNA levels to substantiate the notion.

## Discussion

Mating activates diverse physiological processes for egg laying in insects. One of the critical processes is to stimulate the oviduct activity facilitating egg transport from the ovary to the uterus since anomalies in this activity lead to infertility. The major insect monoamine OA is an important neuromodulator for ovulation but the underlying mechanism is not yet fully understood. In this report we have demonstrated that the G-protein coupled receptor Octβ2R in the oviduct epithelium is essential for ovulation in *Drosophila*. The Octβ2R’s role in ovulation is physiological, rather than developmental, which is consistent with the finding that feeding OA to the mated *tβh* mutant females rescues sterility [14]. We previously showed that another OA receptor, OAMB, located in the oviduct epithelium is indispensable for ovulation as well. Thus, OA acts on both alpha1-like OAMB and beta-like Octβ2R to stimulate the oviduct activity critical for ovulation. Since the females with a homozygous mutation in either *octβ2r* or *oamb* are sterile, individual Octβ2R or OAMB function in the oviduct epithelium is necessary, but not sufficient, to mediate OA’s effects on ovulation.

OAMB activates CaMKII, but not PKA, for ovulation since ectopic expression of constitutively active CaMKII, but not PKA, fully reinstates ovulation in the *oamb* mutant [22]. In contrast, Octβ2R involves both PKA and CaMKII as downstream signaling molecules. OAMB-K3 stimulates both cAMP and Ca^2+^ increases in transfected cells [19] and *in vivo* [22], [25]. Since PKA and CaMKII partially rescue the *octβ2r*’s sterility phenotype, we predicted that OAMB-K3 would offer complete or better rescue than OAMB-AS that increases only Ca^2+^. Ectopic OAMB-K3 expression, however, led to incompletely rescued ovulation like ectopic OAMB-AS expression and to a lesser extent than ectopic Octβ3R expression (Figure 5A). This supports the notion that Octβ2R recruits additional effector systems that Octβ1R, but not OAMB-K3 or Octβ3R, can activate for full fecundity. Given that multiple effectors and signaling pathways are recruited for full fecundity, OAMB-AS, OAMB-K3 and Octβ3R may activate only a subset of effectors or signaling pathways that could support ovulation to a limited extent but are insufficient to execute successful egg laying or progeny production.

**Figure 7:**
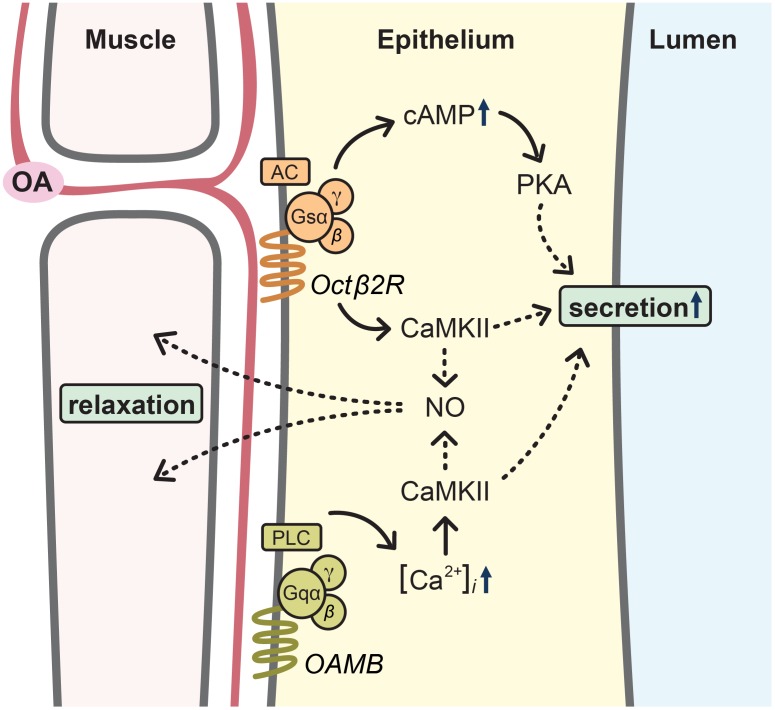
Working model of ovulation mechanism. Mating activates the OA neurons that project to the oviduct epithelium. Binding of OA to the G-protein coupled receptors Octβ2R and OAMB in the epithelium induces cellular activity critical for egg transport from the ovary to the uterus. Specifically, CaMKII activated by Octβ2R together with OAMB may act on nitric oxide synthase (NOS) to release NO, which diffuses to the muscle for relaxation. Concerted activities of PKA and CaMKII activated by Octβ2R, on the other hand, trigger downstream effectors to secrete fluid for egg activation and transport.

There is no information regarding which downstream molecules of PKA and CaMKII or additional cellular components in the oviduct epithelium are involved in the regulation of the oviduct activity for egg transport. OA relaxes the oviduct muscle when applied to the reproductive system [13], however the OA receptor responsible for this action has not been found in the muscle. Thus, it is tempting to speculate that OA activates Octβ2R and OAMB in the epithelium positioned between the visceral muscle and lumen for dual physiological processes, i.e. oviduct muscle relaxation and fluid secretion to the lumen (Figure 7). CaMKII activated by OAMB together with Octβ2R may act on nitric oxide synthase (NOS) to release NO, which in turn travels to the muscle for relaxation in a similar mechanism known in other systems [33]–[36]. This is supported by the observation that NOS knockdown in the oviduct epithelium significantly reduces ovulation (our unpublished data). On the other hand, concerted activities of PKA and CaMKII along with other effectors activated by Octβ2R may be important for fluid secretion to create a suitable chemical environment for egg activation and transport (Figure 7). This is consistent with the finding that ectopic OAMB-AS expression leads to partially restored ovulation but not to progeny production. Mating induces remodeling of the oviduct epithelium to a fully differentiated morphology [37]. It is possible that Octβ2R signaling may be involved in the remodeling process. Alternatively, Octβ2R signaling could be critical for physiological activity of the remodeled epithelium. We favor the latter since ectopic activation of PKA and CaMKII in the wild-type or *octβ2r* epithelium does not induce ovulation without mating (data not shown). It would be important to clarify these notions in follow-up studies.

Most studies in the field of female reproduction have focused on oviposition behavior in an attempt to develop a strategy to lure reproductive females for the management of insect pests and vectors. However, little is known about the physiological and cellular mechanisms mediating the oviposition process. The findings reported here improve our knowledge on this understudied yet important area. Many functions of OA are conserved in insects. For example, OA plays a pivotal role in reward-mediated olfactory learning in fruit flies, honeybees and crickets [28], [38]–[41]. Consistently, OA is implicated in oviposition control in cattle ticks, locust and cowpeas [42]–[45] and the counterparts of OAMB and Octβ2R are found in other insects including all other *Drosophila* species, honeybees, silkworms, locust and mosquitoes [24], [46]–[48]. Enhanced understanding of the mechanism by which OA regulates female fertility would thus help design new strategies to manage beneficial and harmful insects. This study has broader implications as well. Norepinephrine and epinephrine, functional counterparts of OA in vertebrates [16], [46], also regulate ovulation in mammals. The adrenergic receptors that norepinephrine and epinephrine activate are found in the human oviduct epithelium although their functions are yet uncharacterized [49]. Nevertheless, beta-adrenergic agonists induce relaxation of the smooth muscle and stimulate fluid production in the isolated human oviduct [50], [51]. Of particular interest is that the medications commonly used for hypertension, asthma and depression target adrenergic systems, implicating their potential side effects on reproduction. This study, therefore, may help understand fertility issues possibly associated with chronic use of adrenergic drugs.
